# Interkingdom Signaling of the Insect Pathogen *Photorhabdus luminescens* with Plants Via the LuxR solo SdiA

**DOI:** 10.3390/microorganisms11040890

**Published:** 2023-03-30

**Authors:** Nazzareno Dominelli, Alice Regaiolo, Leon Willy, Ralf Heermann

**Affiliations:** 1 Institute of Molecular Physiology (imP), Johannes-Gutenberg-University Mainz, Biocenter II, Microbiology and Biotechnology, Hanns-Dieter-Hüsch-Weg 17, 55128 Mainz, Germany; 2 Institute for Biotechnology and Drug Research gGmbH (IBWF), Hanns-Dieter-Hüsch-Weg 17, 55128 Mainz, Germany

**Keywords:** entomopathogenic bacteria, quorum sensing, bacteria–plant interaction, AidA, LuxR-type receptor

## Abstract

In bacteria, group-coordinated behavior such as biofilm formation or virulence are often mediated via cell–cell communication, a process referred to as quorum sensing (QS). The canonical QS system of Gram-negative bacteria uses *N*-acyl homoserine lactones (AHLs) as communication molecules, which are produced by LuxI-type synthases and sensed by cognate LuxR-type receptors. These receptors act as transcriptional regulators controlling the expression of specific genes. Some bacteria harbor LuxR-type receptors lacking a cognate LuxI-type synthases, designated as LuxR solos. Among many other LuxR solos, the entomopathogenic enteric bacterium *Photorhabdus luminescens* harbors a SdiA-like LuxR solo containing an AHL signal-binding domain, for which a respective signal molecule and target genes have not been identified yet. Here we performed SPR analysis to demonstrate that SdiA acts as a bidirectional regulator of transcription, tightly controlling its own expression and the adjacent *PluDJC_01670* (*aidA*) gene in *P. luminescens,* a gene supposed to be involved in the colonization of eukaryotes. Via qPCR we could further determine that in *sdiA* deletion mutant strains, *aidA* is upregulated, indicating that SdiA negatively affects expression of *aidA*. Furthermore, the Δ*sdiA* deletion mutant exhibited differences in biofilm formation and motility compared with the wild-type. Finally, using nanoDSF analysis we could identify putative binding ability of SdiA towards diverse AHLs, but also to plant-derived signals, modulating the DNA-binding capacity of SdiA, suggesting that this LuxR solo acts as an important player in interkingdom signaling between *P. luminescens* and plants.

## 1. Introduction

Like humans or animals, bacteria can communicate with each other to coordinate group behavior. Bacterial communication employs small diffusible signaling molecules in a process designated as quorum sensing (QS) in which the group-coordinated behavior is dependent on population density or quorum [1]. The most common and well-studied QS-based communication in bacteria is the canonical LuxI/LuxR-type communication in Gram-negative bacteria, where acylated homoserine lactones (AHLs) are produced via an autoinducer synthase LuxI and sensed by a cognate LuxR-type receptor when the AHLs exceed a specific threshold concentration. LuxR-type receptors usually consist of a variable N-terminal signal-binding domain (SBD), which binds the signal and is used to classify different LuxR-type receptors upon their signal-binding property. This domain is connected to the C-terminal helix-turn-helix (HTH) DNA-binding domain (DBD), which specifically binds the target promoters and modulates the transcription of respective genes [2,3,4]. Once AHLs bind to the LuxR-type receptor, the protein exhibits higher affinity towards the respective target promoters; therefore, it is constantly affecting the expression of the respective genes. Furthermore, a positive feedback loop that occurs as transcription of the cognate *luxI* gene is also regulated by LuxR upon signal binding, leading to excessive production of AHLs, for which reason these molecules are also designated as autoinducers [1,5,6,7]. Many proteobacteria harbor LuxR-type receptors; however, some of them lack a cognate LuxI synthase. Such LuxR homologs are designated as LuxR orphans or solos and are widespread among proteobacteria [8,9,10]. Many enteric bacteria such as *Escherichia coli* and *Salmonella enterica* as well as plant-associated bacteria such as *Pseudomonas fluorescens*, *Xanthomonas campestris*, or *Agrobacterium tumefaciens* contain LuxR solos [8]. These LuxR solos can be subgrouped into five classes, class A-E, having different origins, ligands, and ecological roles [11]. Some of them belong to non-AHL-producing bacteria and can sense exogenous AHLs or hormone-like signal molecules produced by bacteria or eukaryotes [10]. SdiA is a LuxR solo found in *E. coli* and *S. enterica* harboring an AHL signal-binding domain but without a cognate LuxI synthase and was therefore suggested to bind exogenous AHLs [12]. Recent docking studies revealed the ability of SdiA to bind long-chain AHLs with higher affinity compared with short-chain AHLs [13].

LuxR solos with a putative AHL-SBD are also found in enteric *Photorhabdus* species, i.e., among the 40 LuxR solos found in insect pathogenic *P. luminescens* ssp. *laumondii* DJC (recently renamed as *P. laumondii* [14]) there are two examples: PluR and SdiA [15,16]. However, for PluR, former studies have revealed a modification in the SBD that leads to perception of endogenous α-pyrones instead of AHLs as signals representing a novel type of cell–cell communication circuit [17]. In contrast, for SdiA in *P. luminescens*, no signal molecule has yet been identified. So far it is known that the SBD of *P. luminescens* SdiA shares high homology with SdiA from other bacteria and contains the conserved amino acid motif (WYDPWG) necessary for AHL binding [16]. For similar LuxR-type receptors, which are widely distributed in plant-associated bacteria such as pseudomonads*,* a possible sensing of AHL-like signaling molecules produced by plant hosts was suggested [18,19]. *P. luminescens* is an entomopathogenic bacterium that undergoes phenotypic switching. While *P. luminescens* primary cells (1°) live in symbiosis with *Heterorhabditis bacteriophora* nematodes, the secondary cells (2°) cannot interact with the nematodes anymore. Both cell forms are genotypically equal, revealing that the phenotypic differences are due to true phenotypic heterogeneity [20]. They appear in the infective life cycle within the insect when all the nutrients from the host cadaver are depleted. Since they cannot reassociate with the nematodes, they remain in the soil when the nematodes have left the depleted insect cadaver and interact with plant roots in the rhizosphere protecting them from phytopathogenic fungi [21,22,23]. To shed light on the role of SdiA in *P. luminescens* and its possible role as interkingdom receptor, we first investigated the effect of SdiA on specific phenotypes such as motility or biofilm formation. Surface plasmon resonance spectroscopy (SPR) was used to reveal the DNA-binding capacity of SdiA towards the promoters of *sdiA* and adjacent *aidA*, an interaction that was affected in presence of plant root exudates. Nano differential scanning fluorimetry (nanoDSF) analyses suggested putative signaling molecules binding towards SdiA, such as AHLs as well as plant compounds. In a quantitative RT-PCR, the expression of *aidA* was revealed to be influenced by the presence and absence of *sdiA*, suggesting an important role for SdiA in *Photorhabdus*–plant interkingdom signaling.

## 2. Materials and Methods

### 2.1. Bioinformatics Analyses

Among the 40 LuxR solos found in the genome of *P. luminescens*, of which two contain an AHL-like signal-binding domain, we investigated the SdiA-like LuxR solo PluDJC_01675, which was highlighted as putative AHL sensor in a previous analysis [16]. A multiple sequence alignment of SdiA with several AHL-LuxR solos from other bacteria was performed using ClustalW [24] to identify sequence motifs in the signal-binding site and the DNA-binding site. Furthermore, a tertiary protein structure of SdiA was predicted using SWISS-MODEL [25,26,27,28,29]. Finally, the BLAST tool was used to search homologous of *aidA* (gene upstream of *sdiA*) in other organisms. Promoter regions of the respective genes *aidA* and *sdiA* were analyzed for *lux*-box-like motifs using Benchling (https://www.benchling.com, accessed on 20 January 2022).

### 2.2. Bacterial Strains, Generation of Deletion Mutants and Expression Vectors

In this study, *Photorhabdus luminescens* DJC 1° and 2° wild-type and respective mutants were used [30]. Deletion mutants of *PluDJC_01675* (*sdiA*) were obtained through *in-frame* deletion via double homologues recombination. For that purpose, ~500 bp fragments up- and downstream of the desired gene were cloned into pNPTS138-R6KT [31] suicide vector using primer pairs FA_*sdiA*_fwd_EagI (catCGGCCGATGAATATTAATCGACCATATGCC) + FA_*sdiA_*rev_**ovl**_FB (**CCTGAGCTT**TCAGCACAGGCCGGAAATTTAGAAC) for flank A and FB_*sdiA_*fwd_**ovl**_FB (**AAGCTCAGG**CCAGGCAATAGCTAAAGCTG) + FB_*sdiA*_rev_SalI (cctGTCGACCCCAAGCTCTGGAAGAATTCCCAT) for flank B for deletion of *sdiA*. Both flanks were fused and inserted into pNPTS138-R6KT using the respective restriction sites. Finally, the obtained vector was transferred into *P. luminescens* 1° and 2° cells via conjugation using *E. coli* ST18 strain [31,32]. *E. coli* BL21 (pLysS) strain was used for heterologous expression of *sdiA*. For that purpose, recombinant pBAD24-N-6xHis-*sdiA* vector*,* in which *sdiA* expression is under control of the arabinose (*ara*) inducible promoter [33], was generated by PCR using primers *sdiA*-N-**6xHis**_fwd_XmaI (gcgCCCGGGATG**CATCATCACCACCACCAT**AATATTAATCGACCATATGCCTTA) + *sdiA*_rev_XbaI (gctTCTAGATTATATATAGCCAAGTAATACAGCTT) and genomic *P. luminescens* DJC DNA as template, and the obtained PCR product was ligated into pBAD24 using respective restriction sites. Bacterial cultures were inoculated into LB medium (1% [*w*/*v*] tryptone, 0.5% [*w*/*v*] yeast extract, 1% [*w*/*v*] NaCl) supplemented with or without the respective antibiotics and aerobically cultivated at 30 °C (*P. luminescens*) or 37 °C (*E. coli*), respectively. If designated, kanamycin was added with a final concentration of 60 µg/mL and carbenicillin with 100 µg/mL.

### 2.3. Motility and Biofilm Assays

To test whether LuxR solo SdiA is involved in modulation of motility or in biofilm formation in *P. luminescens*, swimming, twitching, and biofilm assays with the respective deletion mutant (Δ*sdiA*) in *P. luminescens* and the wild-type were performed. For that purpose, *P. luminescens* was grown overnight and the OD_600_ was adjusted for the respective assay. For swimming motility, 10 µL of an overnight culture with an OD_600_ = 0.1 was spotted in the center of swimming agar plates (0.3% [*w*/*v*] agar, 1% [*w*/*v*] tryptone, and 0.3% [*w*/*v*] NaCl) and incubated for 24 h at 30 °C. The resulting swimming halo diameter was measured using ImageJ (https://imagej.net/ij/index.html, accessed on 15 February 2022). For twitching motility, 10 µL of an overnight culture with an OD_600_ = 0.1 was spotted between the twitching agar (2% [*w*/*v*] agar, 1% [*w*/*v*] tryptone, and 0.3% [*w*/*v*] NaCl) and the petri dish by stabbing the pipette tip through the agar. Plates were incubated for 24 h at 30 °C. Then, the agar was removed from the petri dish, the plates were quickly washed with water, and after drying stained with 1% (*w*/*v*) crystal violet and incubated for 30 min. Afterwards, the plate was washed twice with water and dried overnight. The following day, twitching motility on the surface became visible as bacteria attached on the surface of the petri dish were stained by crystal violet. For quantification of biofilm production, a biofilm assay was performed [30,34,35,36]. Overnight cultures of *P. luminescens* were adjusted to an OD_600_ of 0.5 in LB and 135 µL per well of bacterial suspension was pipetted into transparent polystyrene 96-well microtiter plates and incubated for 24 h and 72 h, respectively, at 30 °C under static conditions. The wells were supplemented with or without PRE to analyze the effect of PRE on biofilm formation of *P. luminescens*. After incubation, the quantification occurred according to O’Toole, 2011 monitoring the absorbance at 575 nm using Tecan Spark plate reader (Tecan Group Ltd., Männedorf, Switzerland).

### 2.4. Extraction of Plant Root Exudates (PREs)

Plant root exudates were collected as described before [22]. *Pisum sativum* variant *arvica* (Bayrische Futtersaatbau, Ismaning, Germany) were grown at 24 °C; 16 h light/8 h dark for 2 weeks in vermiculite. A total of 75 plants were collected, washed, and put into vessels with 250 mL sterile ddH_2_O (for hydrophilic compounds) or methanol (for lipophilic compounds) under continuous shaking for 16 h. The solutions were then filter sterilized and stored at 4 °C in the dark until use.

### 2.5. Heterologous Overexpression of SdiA and Purification of 6His-SdiA

Heterologous expression of *sdiA* was performed in recombinant *E. coli* BL21 pLysS strain carrying the vector pBAD24-N-6xHis-*sdiA*. The 6xHis codon was N-terminally added to the gene via PCR and the resulting PCR product was inserted downstream of the P*_ara_* in expression vector pBAD24. For protein production, an overnight culture of *E. coli* BL21::pBAD24-N-6xHis-*sdiA* cells was prepared and 1 l of LB medium supplemented with the respective antibiotic was inoculated at an OD_600_ = 0.1 and incubated at 37 °C under gentle stirring at 150 rpm until the culture reached an OD_600_ of 0.4. Then, gene expression was induced by adding 0.1% (*v*/*v*) *L*-arabinose to the culture and bacteria were further aerobically cultivated at 30 °C for 3 h. Cells were then harvested by centrifugation for 30 min at 4500 rpm at 4 °C whereupon the pellet was resuspended in lysis buffer (50 mM Tris/HCl, 300 mM NaCl, 5 mM imidazole, 0.5 mM PMSF, 2 mM DTT, pH 7.5). Cells were disrupted by performing three cycles in a French press cell disrupter at 1.35 kbar and cell debris was removed by low-speed centrifugation at 4500 rpm at 4 °C for 15 min. Afterwards, the cytosolic fraction was obtained via ultracentrifugation at 45,000 rpm and 4 °C for 45 min. Then, the cytosol fraction (supernatant) was incubated under gentle shaking at 4 °C with Ni^2+^-NTA-Agarose beads (Qiagen, Hilden, Germany) for purification. After 1 h of incubation, the bead–protein solution was loaded onto a column and the beads were washed twice using 15 mL washing buffer (50 mM Tris/HCL pH 7.5, 10% glycerol, 300 mM NaCl, 40 mM Imidazole). For the second washing step, the imidazole concentration was increased to 80 mM. The 6His-SdiA was eluted using elution buffer (50 mM Tris/HCL pH 7.5, 10% glycerol, 300 mM NaCl, and 250 mM Imidazole), where 6 × 500 µL of the protein solution were collected. To check for successful SdiA production, SDS-PAGE [37] and Western blot analysis using rabbit anti-His antibody (rabbit monoclonal, clone RM146, Sigma Aldrich, Deisenhofen, Germany) and anti-rabbit antibody (anti-rabbit lgG [whole molecule]—alkaline phosphatase antibody produced in goat, Sigma Aldrich) were performed.

### 2.6. Nano Differential Scanning Fluorimetry (nanoDSF)

NanoDSF is a microscale label-free method for rapid and easy detection of protein stability using the intrinsic aromatic amino acids tryptophane and tyrosine to determine protein folding and stability [38,39]. We used the nanoDSF technique to analyze both the stability of purified SdiA and putative binding of several compounds such as AHLs (10 nM C_4_-AHL, 10 nM C_8_-AHL, and 10 nM C_12_-AHL), 3.3% (*v*/*v*) PRE and their respective HPLC-separated fractions to SdiA via protein stability, assuming that ligand binding affects melting temperature of the protein. For that purpose, the protein sample was adjusted to a concentration of 0.3 mg/mL and loaded into capillaries, which were placed into a Prometheus NT.48 (NanoTemper Technologies GmbH, München, Germany) device. The measurement was performed in a temperature range between 20 °C and 90 °C with a temperature slope of 1.5 °C/min. The resulting data were analyzed using the PR.ThermControl software (NanoTemper).

### 2.7. Surface Plasmon Resonance (SPR) Spectroscopy

SPR analysis allows real-time detection of different types of biomolecular interactions, where bindings, specificities, kinetics, and affinities can be determined. Here, we performed SPR analysis using Biacore T200 (Cytiva LifeSciences, Freiburg, Germany) and precoated SA sensor chips (Cytiva), where streptavidin is covalently attached to a carboxymethyl dextran matrix. To test whether SdiA binds to the selected promoter regions, respective DNAs fragments were 5′biotinylated and amplified via PCR from *P. luminescens* DJC gDNA. To achieve ~180 bp fragments of each, [Btn]-P*_sdiA_*, [Btn]-P*_aidA_*, and [Btn]-P*_fliE_*, respective primer pairs [Btn]-P*_sdiA_* fwd + P*_sdiA_* rev, [Btn]-P*_aidA_* fwd + P*_aidA_* rev, and [Btn]-P*_fliE_* fwd + P*_fliE_* rev were used (Table 1). Chip equilibration occurred by injection of 90 µL 1 M NaCl/50 mM NaOH at a flow rate of 10 µL/min for three times. Then, 40 nM of the respective biotinylated promoters were injected with a contact time of 420 s at a flow rate of 10 µL/min and immobilized onto the SA chip. The DNA fragments were diluted in HBS-EP+ buffer (0.01 M HEPES pH 7.4, 0.15 M NaCl, 3 mM EDTA, 0.005% *v*/*v* Tween-20, filtered and degassed) with 0.5 M NaCl. The first flow cell of the chip was kept free and used to obtain a blank sensorgram for subtraction of bulk refractive index background for data evaluation. Then, different concentrations (1.5625 nM, 3.125 nM, 6.25 nM, 12.5 nM, 2 × 25 nM, 50 nM, 100 nM, 200 nM) at a final volume of 150 μL for each dilution of SdiA were prepared in HPS-EP+ buffer for SPR analysis. Additionally, SdiA binding properties to the different promoters were tested under the influence of 3.3% (*v*/*v*) PRE and the respective controls with 3.3% (*v*/*v*) methanol. The run started with an injection time of 180 s, a flow rate of 30 µL/min with a dissociation time of 420 s. Between every cycle the chip surface was regenerated by two regeneration steps, first injecting 2.5 M NaCl, for 60 s at a flow of 30 µL/min followed by an injection of 0.5% (*w*/*v*) SDS for 60 s at a flow rate of 30 µL/min. The resulting sensorgrams were recorded using the Biacore T200 control software 3.2.1 and analyzed with the Biacore T200 evaluation software 3.2.1 (Cytiva) to determine the binding kinetics parameter as well as the binding affinity of SdiA to the tested promoters using a 1:1 binding algorithm.

### 2.8. Real-Time qPCR (RT-PCR)

To investigate the influence of SdiA on the expression of *aidA* real-time qPCR was performed by comparing the expression level of the respective genes of *P. luminescens* 2° Δ*sdiA* with the wild-type. For that purpose, from overnight cultures of both wild-type and deletion mutant, 50 mL cultures with an OD_600_ of 0.05 were prepared and aerobically cultivated for 24 h at 30 °C. In total, three independent biological replicates were sampled, and the total RNA was extracted via the Aqua/Phenol-chloroform-isoamyl alcohol [21] and quality checked using the NanoDrop *One* (Thermo Fisher Scientifics, Waltham MA, USA). Synthesis of cDNA occurred using SuperScript III reverse transcriptase (Invitrogen, Waltham MA, USA) and qPCR followed using specific primers (Table 1). The gene *rpoD* was used as a housekeeping gene [22] and expression of *fliE* was also checked as negative control to confirm SPR data. Pfaffl and Simone equations were used to examine the relative expression values of the target genes and the standard error [40,41], while primer efficiencies were calculated with the LinRegPCR program (http://LinRegPCR.nl, accessed on 8 February 2023).

### 2.9. Preparative High-Performance Liquid Chromatography (HPLC)

To fractionate and enrich the putative plant-derived signal molecules binding to SdiA, the PRE (20 mg/mL in acetonitrile) were fractionized into 48-well plates via preparative HPLC on an Agilent LC system (Santa Clara, CA, USA) using LiChrospher 100 RP18 (125 × 4 mm, 5 μm) column at 40 °C. A linear gradient starting from 1% (*v*/*v*) acetonitrile to 99% (*v*/*v*) acetonitrile in 25 min and then maintaining 100% (*v*/*v*) acetonitrile for 3 min was used at a flowrate of 1 mL/min. The injection volume of the sample was 20 µL/run. Plates were dried to remove residual acetonitrile and stored at −20 °C until further use.

## 3. Results and Discussion

### 3.1. Structural Properties of SdiA

**Figure 1:**
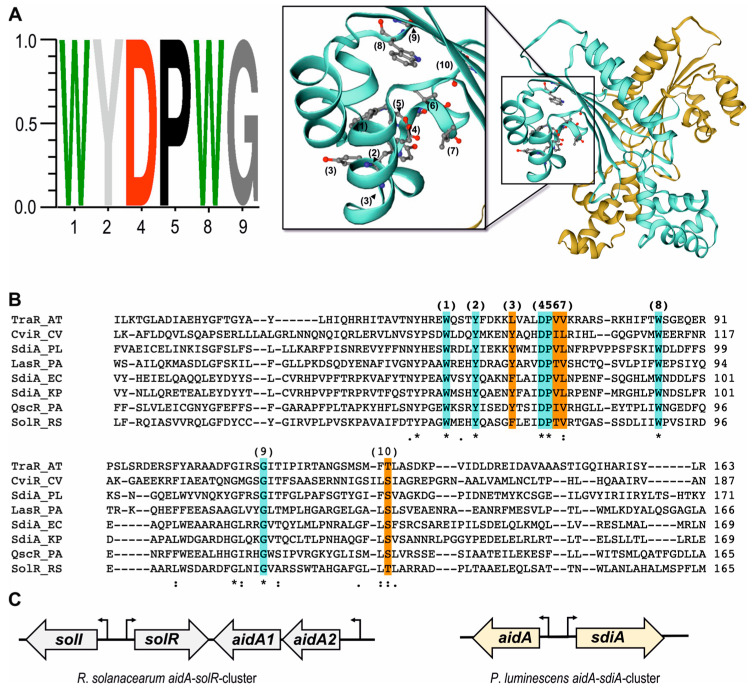
Protein sequence analysis of AHL-LuxR solo SdiA (PluDJC01675) of *P. luminescens*. (**A**) Left panel: sequence logo motif of the six conserved amino acids (AAs) found in AHL-LuxR receptors made with WebLogo3 [49]. Right panel: SdiA tertiary structure predicted with SWISS-MODEL [25,26,27,28,29], pointing out the signal-binding pocket of SdiA. The numbers 1, 2, 4, 5, 8, and 9 below the logo indicate the position of the six conserved amino acids (WYDPWG) in the SdiA model essential for AHL binding. (**B**) Structure-based multiple sequence alignment of the protein sequences of the signal-binding domains (SBDs) of AHL-LuxRs TraR (B9K461) from *Agrobacterium tumefaciens* (ATs), CviR (Q7NQP7) from *Chromobacterium violacaeum* (CV), SdiA (Q7N9K5) from *P. luminescens* (PL), LasR (P25084) from *Pseudomonas aeruginosa* (PA), SdiA (P07026) from *E. coli* (EC), SdiA (A0A0H3GS53) from *Klebsiella pneumoniae* (KP), QscR (G3XD77) from *P. aeruginosa* (PA), and SolR (P58590) from *Ralstonia solanacearum* (RS). The six conserved AAs are highlighted in cyan. In orange are the variable AAs essential for AHL binding of the proteins. The numbers 1 to 10 indicate all AAs essential for AHL binding of *P. luminescens* SdiA in the SWISS-MODEL displayed above. The asterisk indicates fully conserved amino acid residues at the respective position within the alignment. The colon indicates conserved amino acid substitutions with strongly similar prop-erties and a score > 0.5. The period indicates semiconserved amino acid substitutions with weakly similar properties and a score ≤ 0.5. (**C**) Genetic loci of *luxR-aidA* cluster found in insect pathogenic and plant-associated *P. luminescens* and plant pathogenic *R. solanacearum*.

SdiA is a LuxR family transcriptional regulator with a proposed N-terminal AHL signal-binding domain (SBD) occurring in Gram-negative bacteria such as *E. coli* [42] and is also found in entomopathogenic *P. luminescens* [16]. Although SdiA of *P. luminescens* harbors the six conserved amino acid (AA) WYDPWG-motif (Figure 1A) [16] essential for binding AHLs, a respective signaling molecule has not yet been identified. Therefore, the SdiA protein sequence was analyzed and compared with other AHL-LuxR solos occurring in different bacteria. Indeed, the SBD of SdiA harbors in total 10 AA important for binding AHLs. Throughout the clustal alignment analysis the conserved WYDPWG-motif can be observed occurring in all analyzed AHL-LuxR receptors shaping the basic structure of the ligand-binding pocket (Figure 1A,B [marked in cyan]). However, the other 4 AAs in the essential part of the SBD vary between the different LuxR receptors (Figure 1B (highlighted in orange)), very likely resulting in an altered shape of the ligand-binding pocket, making the specificity towards different signal molecules [19,43]. These varieties also occur between the SdiA of different organisms, i.e., at position (3) (Figure 1B) Tyr73 of SdiA in *P. luminescens* is substituted with Phe76 in *E. coli*. Interestingly, variations in these regions in LuxR receptors of plant-associated bacteria were reported, whereupon these differences suggested specificity towards different molecules including plant compounds [19,44,45]. *P. luminescens* harbors a gene upstream of SdiA coding for PluDJC_01670, a one-domain protein harboring a PixA domain, similar to AidA2, found in plant pathogenic *Ralstonia solanacearum*. Interestingly, *R. solanacearum* harbors two AidA encoding genes (here named *aidA2* and *aidA1*), both located upstream of *solR*, which codes for an AHL-LuxR receptors strongly regulating the expression of both *aidA* genes [46,47]. Although the orientation of *aidA* and *sdiA* in *P. luminescens* differs from the *aidA*–*solR* cluster in *R. solanacearum*, we suggest regulation of *aidA* expression via SdiA in *P. luminescens* as the genes share an intergenic promoter region (Figure 1C) harboring putative *lux*-box-like motifs (Figure 4A), which are important for DNA-binding of LuxR-type receptors [48]. Furthermore, BLAST analysis revealed similarity of about 27% between AidA of both bacteria, which lends support to the idea that *P. luminescens* AidA might be involved in host, predominantly in plant colonization, similar to that suggested for *R. solanacearum* AidA [47]. For that purpose, we supposed that *P. luminescens* SdiA might be involved in regulation of *aidA* gene and might therefore be involved in bacteria–plant interkingdom signaling.

### 3.2. Influence of SdiA on Motility and Biofilm Formation

**Figure 2:**
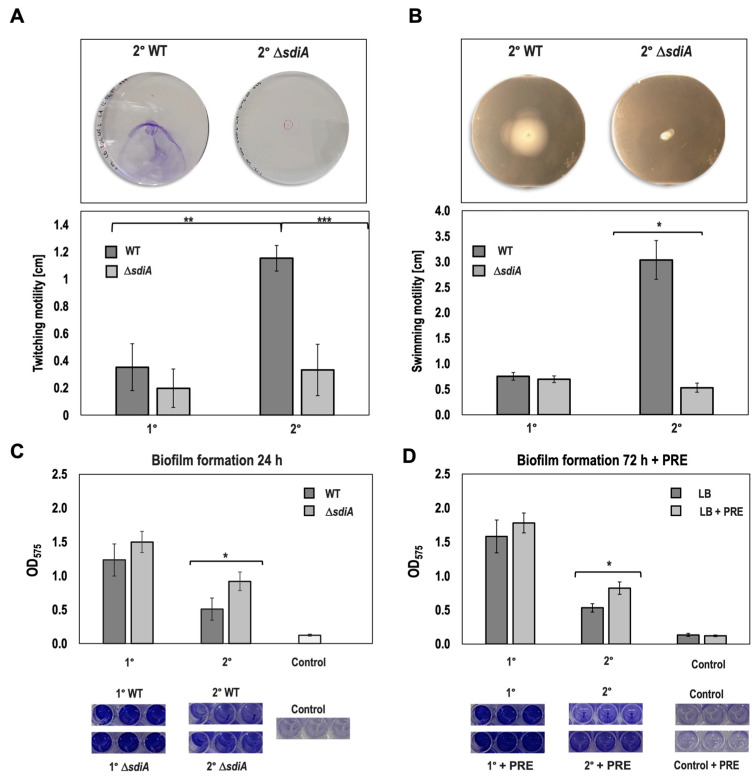
Influence of SdiA on motility, twitching, and biofilm formation of *P. luminescens* 1° and 2° cells. (**A**) Twitching motility: at the top crystal violet staining of twitched cells adhering on the surface. The pictures represent one characteristic of at least three independently performed experiments with similar outcomes. (**B**) Swimming motility of Δ*sdiA* compared with the wild-type (WT). (**C**) Biofilm formation: crystal violet-stained biofilm quantified at 575 nm after 24 h of 1° and 2° cells wild-type (WT) and the respective Δ*sdiA* mutant. Below crystal violet staining. (**D**) Biofilm formation in presence of 3.3% (*v*/*v*) PRE after 72 h of incubation. Below crystal violet staining. The error bars represent the standard deviation of at least three biological replicates. *, **, *** all indicate *p* ≤ 0.05 of comparison between the different groups.

In bacterial mutualism and virulence, not only biofilms, but also motility through swimming and twitching are essential for a successful host colonization. Especially twitching motility, which is a movement driven by pilus extension, attachment, and retraction on viscous or solid surfaces, plays a major role in pathogenesis [50,51]. Indeed, for plant pathogenic *R. solanacearum*, both motility strategies are critical for plant colonization and development of maximal virulence [52,53]. For *P. luminescens*, it is known that 2° cells are highly motile compared with 1° cells and react to PRE, which was suggested to be an important trait for plant colonization [21,22]. However, in the previous study, only swimming and not swarming capacity was investigated. Therefore, we first performed twitching motility assays, showing that 2° cells display significantly higher twitching motility on solid petri dish surface compared with 1° cells (Figure 2A), which might be an important trait that 2° cells use to move in the rhizosphere.

Generally, biofilm formation and motility can be regulated via QS as it was demonstrated for *Vibrio harveyi* [54,55]. Interestingly, LuxR solo SdiA of non-AHL-producing enteric bacteria such as *Escherichia*, *Salmonella*, or *Klebsiella*, influence expression of genes related to virulence factors such as biofilm formation or motility [56,57,58,59,60,61], which is not necessarily mediated upon signal binding [59,62,63,64,65,66]. For that reason, we first analyzed the influence of *sdiA* deletion on swimming and twitching motility as well as biofilm formation of *P. luminescens*. Remarkably, *P. luminescens* 2° Δ*sdiA* deletion mutant showed a totally impaired swimming as well as twitching capacity in comparison with 2° WT (Figure 2A,B), a behavior already observed for other bacteria such as *Vibrio harveyi* and plant pathogenic *Acidovora citrulli*, when deleting genes encoding LuxR receptors [55,67]. Furthermore, Yang and Defoirdt [55], as well as Wang and colleagues [67], also reported increased biofilm formation upon *luxR* deletion, which was also described for pathogenic *Klebsiella* lacking *sdiA* [68], an effect that we can also observe in *P. luminescens* Δ*sdiA*. Indeed, the mutants displayed an increased biofilm formation of about 67% compared with the wild-type in 2° cells, which was significantly visible after 24 h of incubation (Figure 2C, left panel). Based on these results we hypothesize a signal-independent regulatory crosstalk of *P. luminescens* SdiA positively regulating motility, whereas biofilm formation is repressed. It is likely, that SdiA plays a role in regulating a switch between a sessile and a motile lifestyle in *P. luminescens* 2°. Moreover, supplementation of 1° and 2° cells with PRE had the same effect as the deletion of *sdiA* with respect to motility and biofilm formation, which is most predominant in 2° cells. While motility of *P. luminescens* decreased in the presence of PRE [22], biofilm formation increases of about 54% (Figure 2C, right panel); however, this effect was only visible after 72 h. Therefore, from our finding we further propose that the regulatory connection between SdiA and biofilm formation and motility is distinctive in the absence of signaling molecules, but only affected in the presence of PRE. This is in accordance with the finding of the LuxR receptor EsaR in *Pantoea stewartii*, which was only active in the absence of the respective signal molecule [69]. Furthermore, it has been suggested for *E. coli* SdiA that the primary alteration of the protein had more impact on the expression of biofilm-related genes than binding of a signaling molecule. However, a plant-derived indole derivative binds SdiA leading to reduced biofilm formation indicating a negative effect on the regulatory role of SdiA upon signal binding [70,71]. Therefore, we hypothesized that SdiA is involved in interkingdom signaling (IKS) between *P. luminescens* and plants. It is likely that SdiA in its native conformation represses biofilm formation in 2° cells, while activating expression of genes responsible for motility. This regulation is then changed once 2° cells are close to the plants, so that SdiA binds a plant-derived molecule resulting in reduced motility and increased biofilm formation to colonize the new plant host.

### 3.3. Plant Root Exsudates and AHLs Influence Protein Stability Suggesting Putative Signals for SdiA

**Figure 3:**
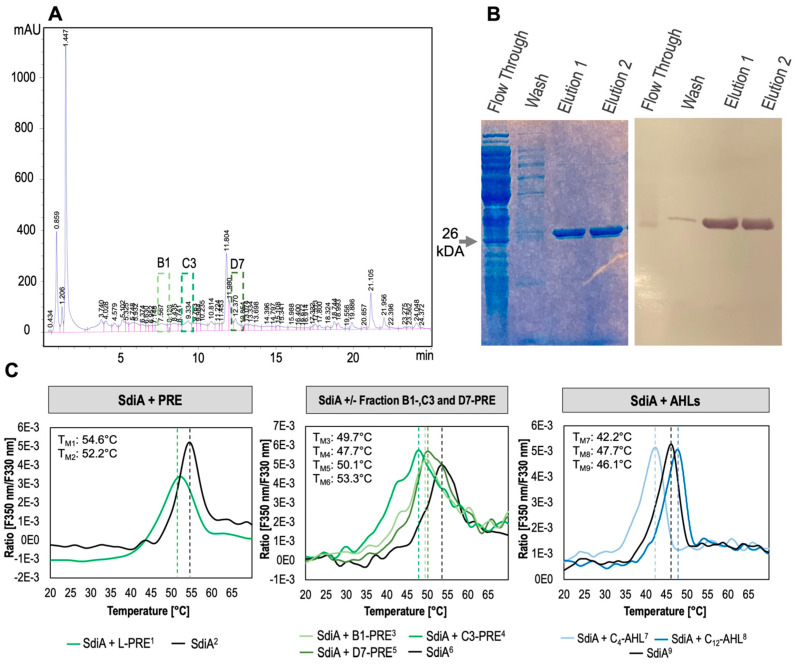
HPLC chromatogram of plant root exudates, SdiA purification, and thermostability of SdiA in the presence of AHLs and PRE. (**A**) Chromatogram of semipreparative HPLC of plant root exudates separated in 48 fractions. The green dashed lines represent the fractions showing where putative signal molecules for SdiA are located. Light green: fraction B1; green: fraction C3; and dark green: fraction D7. (**B**) On the left panel SDS-PAGE representing purification of 6xHis-SdiA, which was confirmed via Western blot analysis (right panel). (**C**) NanoDSF analyses of SdiA with supplementation of lipophilic plant root exudates (L-PRE, left panel), the selected respective HPLC fractions (B1, C3, D7, middle panel) and 10 nM of C_4_- or lll C_12_-AHLs (right panel). The graphs represent the 1st derivative of the measured ratio of intrinsic tryptophane fluorescence at 350 and 330 nm of the protein. The maximum peak represents the melting temperature T_M_, where half of the protein molecules are denatured. T_M_ values are indicated by the dotted lines colored respectively to the curve. In all measurements the black line shows the control protein measured with solvent, when necessary. Left shift of the graphs indicate different protein conformation upon molecule binding. In the left panel: PRE influence SdiA stability upon putative signal molecule binding with DT_M_ −2.4°C. Middle panel: putative signaling molecule found in PRE-fractions indicated by DT_M_ −3.8 °C (B1, light green), DT_M_ −5.8 °C (C3, green), and DT_M_ −3.4 °C (D7 dark green). Right panel: AHLs putatively bind to SdiA with different modes of action indicated by DT_M_ −3.9 °C (C4-AHL, light blue) and DT_M_ +1.6 °C (C12-AHL, blue), showing increased stability of the protein.

To further investigate the correlation observed for motility of *P. luminescens* 2° cells in presence of PRE and the Δ*sdiA* phenotype, the effect of PRE on purified SdiA protein stability was examined. For that purpose, nano differential scanning fluorimetry (nanoDSF) was used (i) to exclude possible buffer-derived denaturing of SdiA during protein purification and (ii) to test the protein stability in the presence of PRE and AHLs. Furthermore, PRE were separated into 48 fractions (Figure 3A) and tested on SdiA. It was described earlier that signal binding to LuxR-like receptors promotes conformational changes or changes in oligomerization [72], so that signal binding should result in differences in thermostability of SdiA. The measurement showed high thermostability of SdiA with an onset point (T_ON_) on average of about 44 °C, where the protein started to unfold. The inflection point, on average at 53.3 °C, indicates the moment where half of the protein appears in an unfolded state and is equal to T_M_ (Figure 3C). Control measurement supplementing SdiA solely with the respective solvent that used to suspend the AHLs to exclude solvent-derived effects on the melting temperature showed no significant effects (SdiA T_M_ was determined as 46.1 °C) (Figure 3C, right panel). Furthermore, we tested the influence of several compounds such as short-chain C_4_-AHL, long-chain C_8_- and C_12_-AHL and PRE, as well as respective HPLC fractions on the protein folding properties in order to identify possible signal molecules recognized by SdiA. A putative ligand binding to LuxR-type receptors leading to conformational changes [72] becomes visible as the temperature shifts on the protein melting temperature T_M_, which can be measured. We observed that both, C_4_- and C_12_-AHL influenced SdiA folding temperature (C_4_-DT_M_= −3.9 °C and C_12_-DT_M_= +1.6 °C). However, upon binding of C_12_-AHL, thermostability of SdiA increased appearing as a ‘right-shifted’ curve with higher T_M_ (47.7 °C). Surprisingly, SdiA was less stable in the presence of C_4_-AHL, as indicated by ‘left-shifted’ curve with lower T_M_ (42.2 °C) compared with the control (Figure 3C, right panel). These data indicate a lower selectivity of SdiA towards different AHLs which is likely dependent on the lengths of the acyl chain. The signal-binding pocket of the receptor probably appears in different conformational states, upon signal binding, putatively influencing DNA-binding properties of SdiA. Similarly, for *E. coli* SdiA, it was shown that at least derivatives of AHL were also capable to act as folding switch autoinducers for the receptor [73]. Indeed, our data strengthen the hypothesis that the signal independent regulatory role of SdiA is disturbed by signal binding, since we could observe unstable conformations of the protein upon putative signal binding.

Additionally, it is known that plants produce molecules mimicking AHLs, which are sensed by, e.g., *Pseudomonas fluorescens* [18,19]. To gain insights whether SdiA of *P. luminescens* also responds to plant-derived compounds, we tested PRE as full mixture and subsequently as fractions separated through HPLC (Figure 3A). Indeed, our data indicate that a yet unknown compound in PRE binds to SdiA as the protein thermostability was affected, i.e., fractions B1, C3, and D7 decreased the SdiA unfolding temperature (lower T_M_) which was visible as a ‘left-shifted’ curve compared with the SdiA-control (Figure 3C, left and middle panel). Accordingly, we propose that PRE contain putative signal molecules, which are recognized by SdiA of *P. luminescens*. However, the chemical nature remains unclear. At this point various possible inducer molecules produced by plants could come into play. Different studies showed a variety of molecules, different to AHLs, which are produced by plants and bind LuxR regulators. Since we observed that AHLs bind to SdiA of *P. luminescens*, and it was earlier shown that those molecules were found in pea root exudates [74], it might therefore be possible that they are present in the PRE used in this study and influenced the thermostability of SdiA. Further possible candidates of signal molecules binding SdiA are glycerol and respective derivatives, which bound to SdiA of enterohaemorrhagic *E. coli* (EHEC) in the absence of AHLs [66], and that are also present in PRE [22]. Furthermore, plant-derived indole compounds, influenced SdiA-mediated regulation of gene expression in *E. coli* [70]. Interestingly, in recent studies plant-derived ethanolamine derivatives were shown to bind a LuxR-type regulator from *Pseudomonas spec*. that subsequently led to induction of the expression of different genes [45]. Therefore, ethanolamine derivatives should also be considered as putative signals for *P. luminescens* SdiA.

Taken together, we suggest a new communication circuit where SdiA is involved in IKS communication with plants, whereupon SdiA modulation of gene expression might be affected by signal binding due to conformational changes. Thereupon, SdiA of *P. luminescens* can be classified into the class D of the LuxR family regulators, which represent LuxR solos responding to non-AHL exogeneous signals [11]. Our findings suggest that it is likely that the SdiA conformational changes, indicated by the temperature shifts upon binding short-chain AHLs and PRE, result in impaired dimerization of the protein, and putatively in reduced DNA-binding affinity. This is in accordance with the findings for the LuxR-type receptor EsaR of *Pantoea stewartii*, where AHLs were found to block DNA-binding capacity [75,76,77,78]. This would also explain the phenotypic changes regarding biofilm formation and motility for *P. luminescens*, which were similar in the Δ*sdiA* mutant or exposing the respective wild-type to PRE. However, it must be determined, which plant-derived compound(s) bind to SdiA of *P. luminescens*, and whether SdiA-DNA-binding capacity is really affected upon signal binding.

### 3.4. SdiA Binds P_sdiA_ and P_aidA_ with High Affinity and Negatively Affects Expression of AidA

**Figure 4:**
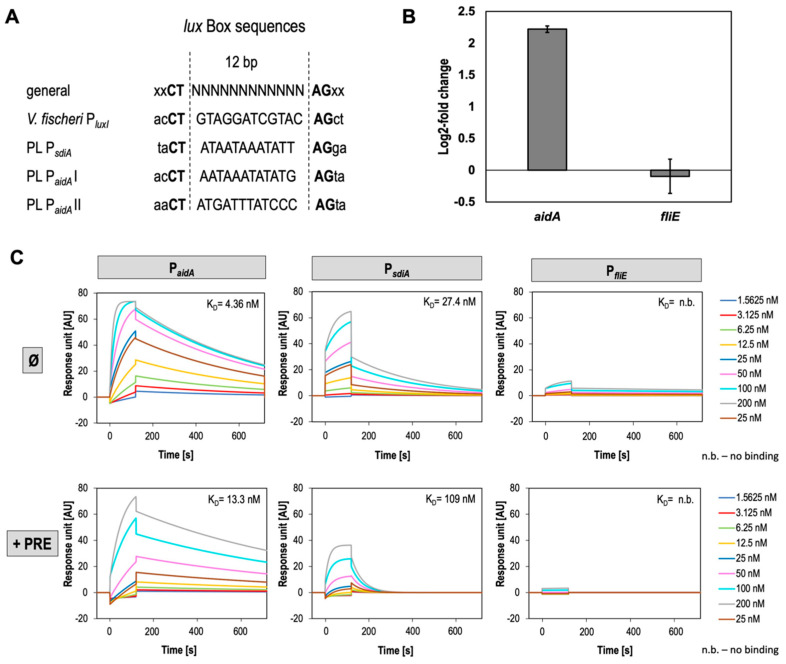
DNA-binding of SdiA to P*_sdiA_*, P*_aidA_,* and P*_fliE_*. (**A**) Putative *lux*-like recognition site in the intergenic region between s*diA* and *aidA* of *P. luminescens* (PL) compared with the *lux*-box motif within the *luxI* gene of *Vibrio fischeri*. PL P*_aidA_*I and II represent two *lux*-boxes found in the promoter region *aidA*. (**B**) Real-time qPCR demonstrating expression of *aidA* and *fliE* (control). The plot shows the log_2_-fold change in *P. luminescens* 2° Δ*sdiA* versus the wild-type. The analysis was performed after incubating the cells for 24 h at 30 °C in LB medium. Error bars represent standard deviation of at least three independently performed biological experiments (*p* ≤ 0.05). (**C**) Binding kinetics of SdiA to the promoters P*_aidA_*, P*_sdiA_*, and P*_fliE_*(negative control) using surface plasmon resonance (SPR) spectroscopy. The respective DNA fragments containing the putative promoter regions were immobilized onto a SA sensor chip and different concentrations of SdiA: 1.5625 nM (iris blue), 3.125 nM (red), 6.25 nM (green), 12.5 nM (yellow), 25 nM (blue and brown), 50 nM (pink), 100 nM (turquoise), and 200 nM (grey) were injected without (top panel indicated by Ø) and with addition of PRE; n.b.: no binding. All graphs and sensorgrams represent one characteristic measurement of at least three independently performed experiments with similar outcome.

It is well-known that transcriptional LuxR-regulators undergo conformational changes upon signal binding enabling the C-terminal HTH domain to bind target gene promoter at the *lux*-box conserved sites to control gene transcription [79,80]. In a prototypical QS circuit, LuxR-type regulators induce transcription of the cognate *luxI*, which is also designated as autoregulation [5]. However, since in *P. luminescens* SdiA is a LuxR solo as the bacteria do not harbor any *luxI* gene, we hypothesized that SdiA might regulate activity of its own promoter region. To validate this idea and the hypothesis that SdiA regulation is signal independent, we first studied the binding affinity of SdiA to its respective promoter (P*_sdiA_*) via surface plasmon resonance (SPR) spectroscopy without any putative ligand. Furthermore, we also analyzed the binding of SdiA towards the promoter of neighboring gene *aidA* (P*_aidA_*) since this gene is located upstream of *sdiA* and encodes in the opposite orientation. Both genes share a 310 bp long promoter containing intergenic region harboring putative *lux*-box-like motifs with a 12 bp long variable motif between with a 5′CT and a 3′AG (Figure 3A) [48,81], putatively necessary for SdiA-binding. As negative control we used P*_fliE_* not containing any *lux*-box-like motif in the respective promoter region (Figure 4C). Remarkably, SdiA showed a high binding affinity towards its own promoter P*_sdiA_* due to a high association rate (*k_a_* = 1.1 × 10^5^/M*s) and a dissociation rate of *k_d_* = 3.1 × 10^−3^/s resulting in an overall affinity of K_D_ = 27.4 nM. This reveals a fast self-regulatory property of SdiA with rapid association and fast dissociation. However, SdiA bound with a ~3.5× higher association rate to the promoter of *aidA* (P*_aidA_*), caused by an extreme low dissociation rate (*k_d_* = 1.7 × 10^3^/s) with comparable association rate to P*_sdiA_* (*k_a_* = 3.9 × 10^5^/M*s), resulting in an overall affinity of 4.4 nM (Figure 4C). This demonstrated a very strong interaction of SdiA with P*_aidA_*, proving our hypothesis that SdiA regulates expression of *aidA* similar to as it was described for the LuxR-type receptor SolR of *R. solanacearum*. SolR was found to positively regulate expression of *aidA* genes and is required for virulence towards plants [47]. Additionally, for the SdiA-P*_aidA_* interaction we can observe a 1:2 binding stoichiometry indicated by a double R_max_ value, which is in accordance with the occurrence of two putative LuxR-binding boxes in the respective promoter region, designated as P*_aidA_*I and P*_aidA_*II (Figure 4A). In contrast, for P*_sdiA_*, only a 1:1 binding stoichiometry could be observed by the SPR analysis indicating only putative one SdiA-binding site. Interestingly, these data suggest that the 310 bp long intergenic region between *aidA* and *sdiA* acts a bidirectional promoter. Thus, SdiA is capable of bidirectional stimulation of transcription of these two oppositely oriented genes. A similar binding mechanism is also described for LuxR of *Vibrio fischeri*, similarly stimulating *lux* operons located in the opposite direction [82].

To elucidate whether DNA-binding ability of SdiA is reduced in the presence of putative signal molecules, the binding properties of SdiA towards P*_sdiA_* and P*_aidA_*were investigated using DNA-binding kinetics also in the presence of PRE. Indeed, the binding affinity of SdiA towards both promoters was reduced. While association towards P*_sdiA_* remained more or less similar, the dissociation from the DNA was observed to be much faster in the presence of PRE (*k_a_* = 1.8 × 10^5^/M*s, *k_d_* = 2.0 × 10^−2^/s) resulting in an overall affinity of 109 nM (Figure 4c bottom left panel). Similarly, a faster dissociation of SdiA from P*_aidA_* occurred in the presence of PRE (*k_d_* = 1.1 × 10^−3^/s), whereas here also the association rate was affected (*k_a_* = 8.2 × 10^4^/M*s), resulting in a lower overall affinity of 13.3 nM, (Figure 4c, bottom middle panel). Solvent-derived effects of PRE on binding kinetics can be excluded, as affinity of SdiA towards the respective promoters was not affected in the presence of the solvent methanol (N.D. and R.H. unpublished). SdiA did not significantly bound to P_fliE_, indicating that the interactions observed for P*_sdiA_* and P*_aidA_* were specific (Figure 4C, bottom right panel). Furthermore, we used real-time qPCR to confirm that SdiA-binding to P*_aidA_* also affects the expression of the corresponding gene. The housekeeping gene *rpoD* was used as control. Indeed, *aidA* expression showed to be negatively regulated by SdiA as we could detect an increased expression of the gene with a log_2_-fold change in 2.22 in the *sdiA* deletion mutant of *P. luminescens* (Figure 4B).

In summary, these data further support the hypothesis that a plant-derived signaling molecule binds to SdiA and subsequently influences DNA-binding of the receptor. Therefore, SdiA acts as LuxR-type receptor that is involved in interkingdom communication (IKS) of *P. luminescens* with plants. However, the molecular mechanism behind the chemical nature of the signaling molecule is unknown. Considering the role of AidA in *R. solanacearum* and the regulation of *aidA* expression mediated by SolR, our data further propose a putative role of AidA in *P. luminescens* in bacterial–plant interaction as *aidA* expression is under control of SdiA and the gene is clustered with *sdiA*, similar as found in *R. solanacearum* (Figure 1B), and as SdiA DNA-binding affinity is affected in the presence of PRE. In *P. luminescens*, it can be supposed that SdiA negatively affects expression of *aidA* in the absence of a signal molecule, and once SdiA senses a plant-derived signal, the affinity towards the promoter of *aidA* is reduced, leading to enhanced expression of the gene putatively important for plant host colonization. The exact molecular mechanism behind this regulation as well as the function of AidA in *Photorhabdus*–plant interaction remains unclear. Hence, further insights into the effect of the putative mentioned signal molecules on SdiA DNA-binding property should be gained, to understand the regulatory hierarchy of SdiA upon plant signals in *P. luminescens*.

## 4. Conclusions

**Figure 5:**
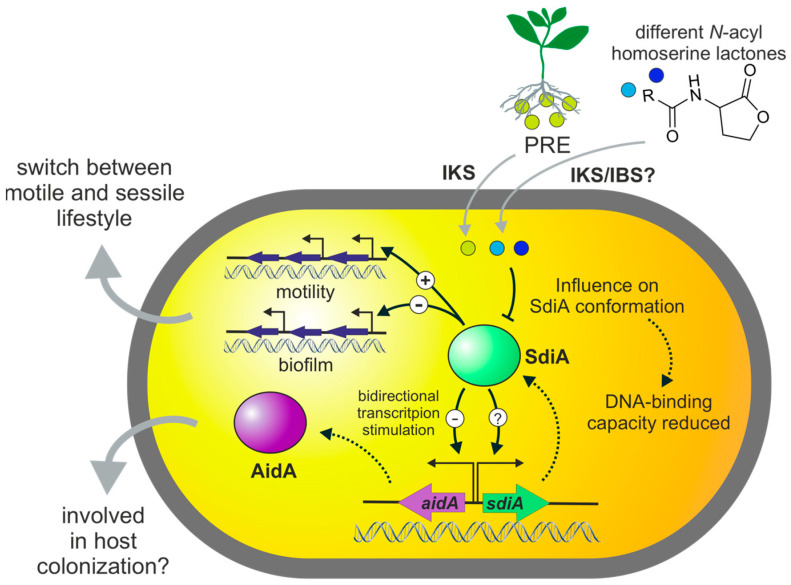
Model of the putative role of AHL-LuxR solo SdiA of *P. luminescens*. The LuxR-type receptor SdiA (PluDJC_016750) regulates the expression of several genes in absence of a signaling molecule, thereby modulating a switch of *P. luminescens* cells between a sessile and motile lifestyle. SdiA is involved in interkingdom signaling (IKS) communication with plants. After sensing a putative plant-derived signal, the protein undergoes conformational changes resulting in reduced DNA-binding capacity. SdiA acts as bidirectional regulator of transcription, binding within the intergenic region of *sdiA* and *aidA* genes. The regulation of *aidA* is negatively affected upon SdiA binding, which might be offset upon binding plant signals. AidA is therefore supposed to be involved in SdiA-mediated host colonization. However, SdiA reacts to different AHLs, thereby it might also be involved in interbacterial signaling (IBS); therefore, upon communication with neighboring AHL-producing bacteria, the expression of SdiA-controlled genes might be affected in a bacterial cell community.

Recent research showed potential biocontrol ability of *P. luminescens* 2° cells since the bacteria can colonize plant roots, protecting them from phytopathogenic fungi infection. As this cell variant does not re-associate with nematodes and interacts with plants, it was important to understand and to determine how the bacteria sense the rhizosphere environment. We can demonstrate that the LuxR solo SdiA harboring an AHL-like SBD is involved in IKS communication between *P. luminescens* and plants, potentially involving AidA in plant host colonization. First, in this work we demonstrated a regulatory crosstalk between SdiA motility as well as biofilm formation regulating a putative switch between a sessile and motile lifestyle of the bacteria (Figure 5). Nevertheless, which other genes beside *aidA* and *sdiA* are under control of SdiA is unknown and must be determined. From our findings we conclude that SdiA regulates expression of *aidA* and *sdiA* in a signal-independent manner (Figure 5). Here, we demonstrated that SdiA protein conformation is affected by short-chain AHLs and PRE and respective fractions indicating (i) plant-derived molecule(s) as signal for SdiA and (ii) an influence of signal binding on the regulatory role of SdiA. Although the nature of the plant-derived compound(s) must be determined, the data suggest a SdiA-mediated interkingdom communication of *P. luminescens* with plants as DNA binding was also influenced upon PRE. We also identified that SdiA acts as a bidirectional regulator binding in the intergenic region of *sdiA* and *aidA* since the receptor binds both promoters with high affinity, most probably negatively affecting expression of *aidA*. Lastly, for AidA of *P. luminescens*, of which homologs are also found in plant-associated bacteria and are regulated by a LuxR, we suggest an involvement in *P. luminescens* plant colonization whereby SdiA controls the expression of *aidA* depending on IKS with the plant host (Figure 5).

## Figures and Tables

**Table 1:** Oligonucleotides used for amplification of biotinylated DNA for SPR analysis and real-time qPCR.

Primer Name	Sequence 5′-> 3′
[Btn]-P*_sdiA_* fwd	[Btn]-GATTATTAGGATTTCAATCCTATTGATAT
P*_sdiA_* rev	TCAATGTCCTCTTGAAAATTAAG
[Btn]-P*_aidA_* fwd	[Btn]-GACACCTCTTTACATATTTAAACTATT
P*_aidA_* rev	CTATATGAAGCAATACCTAATAAATATATG
[Btn]-P*_fliE_* fwd	[Btn]-GTCATTATTCGCTGTTCACTC
P*_fliE_* rev	AAAAACCTCGTGTTAAACCAC
*aidA*-qPCR-fwd	TCCAACAGTTATCCGTCAGC
*aidA*-qPCR-rev	GCCCTCCATCTAATATTCGCA
*fliE*-qPCR-fwd	GTGCTGCAACTGATGCAAG
*fliE*-qPCR-rev	GAGCTCGTTTTGTGGCATTC
*rpoD*-qPCR-fwd	CGGAAGATATCGTCGATTCCGA
*rpoD*-qPCR-rev	TGTCGTTAGCGGTTTCTGCT

## Data Availability

Data is contained within the article or available on request from the corresponding author.
